# Transgenic mice overexpressing desmocollin-2 (DSC2) develop cardiomyopathy associated with myocardial inflammation and fibrotic remodeling

**DOI:** 10.1371/journal.pone.0174019

**Published:** 2017-03-24

**Authors:** Andreas Brodehl, Darrell D. Belke, Lauren Garnett, Kristina Martens, Nelly Abdelfatah, Marcela Rodriguez, Catherine Diao, Yong-Xiang Chen, Paul M. K. Gordon, Anders Nygren, Brenda Gerull

**Affiliations:** 1 Department of Cardiac Sciences and Libin Cardiovascular Institute of Alberta, University of Calgary, Calgary, Alberta, Canada; 2 Schulich School of Engineering, University of Calgary, Calgary, Alberta, Canada; 3 Alberta Children's Hospital Research Institute Genomics and Bioinformatics Facility, University of Calgary, Calgary, Alberta, Canada; 4 Comprehensive Heart Failure Center and Department of Internal Medicine I, University Hospital Würzburg, Würzburg, Germany; Texas A&M University Health Sciences Center, UNITED STATES

## Abstract

**Background:**

Arrhythmogenic cardiomyopathy is an inherited heart muscle disorder leading to ventricular arrhythmias and heart failure, mainly as a result of mutations in cardiac desmosomal genes. Desmosomes are cell-cell junctions mediating adhesion of cardiomyocytes; however, the molecular and cellular mechanisms underlying the disease remain widely unknown. Desmocollin-2 is a desmosomal cadherin serving as an anchor molecule required to reconstitute homeostatic intercellular adhesion with desmoglein-2. Cardiac specific lack of desmoglein-2 leads to severe cardiomyopathy, whereas overexpression does not. In contrast, the corresponding data for desmocollin-2 are incomplete, in particular from the view of protein overexpression. Therefore, we developed a mouse model overexpressing desmocollin-2 to determine its potential contribution to cardiomyopathy and intercellular adhesion pathology.

**Methods and results:**

We generated transgenic mice overexpressing DSC2 in cardiac myocytes. Transgenic mice developed a severe cardiac dysfunction over 5 to 13 weeks as indicated by 2D-echocardiography measurements. Corresponding histology and immunohistochemistry demonstrated fibrosis, necrosis and calcification which were mainly localized in patches near the epi- and endocardium of both ventricles. Expressions of endogenous desmosomal proteins were markedly reduced in fibrotic areas but appear to be unchanged in non-fibrotic areas. Furthermore, gene expression data indicate an early up-regulation of inflammatory and fibrotic remodeling pathways between 2 to 3.5 weeks of age.

**Conclusion:**

Cardiac specific overexpression of desmocollin-2 induces necrosis, acute inflammation and patchy cardiac fibrotic remodeling leading to fulminant biventricular cardiomyopathy.

## Introduction

Arrhythmogenic cardiomyopathy (AC), (also known as arrhythmogenic right ventricular cardiomyopathy, ARVC), is an inherited cardiomyopathy leading to heart failure, arrhythmias and sudden cardiac death often in young people. Fibro-fatty replacement of the myocardium is a typical histological hallmark of AC [1]. Up to 50% of AC patients have one or more mutations in five genes encoding cardiac desmosomal proteins: *JUP* [2], *PKP2* [3], *DSP* [4], *DSC2* [5] and *DSG2* [6].

Cardiac desmosomes are cell-cell junctions connecting the intercalated disc to the intermediate filament system and have important mechanical functions [7]. In addition, there is increasing evidence that desmosomes also have a signaling function within the cell [8].

Desmocollin-2 (Dsc2) and desmoglein-2 (Dsg2) are members of the cadherin family and contribute to the interconnection of cardiomyocytes [9]. The intracellular domains of desmosomal cadherins bind to plakophilin-2 (Ppk2) and plakoglobin (Jup), which are members of the armadillo protein family [9, 10]. Both proteins are linked to desmoplakin (Dsp), a cytolinker protein, which connects desmosomes and desmin filaments [11].

Apart from genetic association between AC and mutant desmosomal genes, molecular and cellular pathomechanisms are poorly understood. Over the last decade several mouse models have been created to get functional insights into the pathogenesis of desmosomal gene alterations. However, embryonic lethality caused by global knock-out of Jup [12, 13], Dsp [14], Dsg2 [15] and Pkp2 [16] demonstrated on one hand the general importance of those desmosomal proteins, but limited on the other hand functional analyses *in vivo*. To circumvent those limitations, several conditional knock-out, knock-in or transgenic mouse models for Dsp [17–19], Dsg2 [20–22], Jup [23] and Pkp2 [16, 24] have been developed, which mimic partially an AC phenotype. In contrast, complete Dsc2 knock-out did not induce a cardiac phenotype in mice under normal housing conditions [25]. However, to our knowledge no other transgenic or mutant Dsc2 knock-in mouse models have been reported.

Here, we demonstrate for the first time that cardiac specific overexpression of DSC2 causes a severe cardiomyopathy in mice. While transgenic mice are healthy at birth, they rapidly develop extensive cardiac fibrosis in combination with necrosis and calcification between the second and third week of post-natal life, leading to decreased ventricular fractional shortening and ejection fraction. Furthermore, these transgenic mice do not experience sudden cardiac death, but show prolonged electrical depolarization likely secondary to the extensive structural impairment of their hearts. Gene expression analysis in combination with immunohistochemistry suggests induction of inflammatory and fibrotic remodelling pathways.

## Material and methods

### Generation of transgenic *DSC2* mice

Human DSC2 cDNA was cloned via *Sal*I and *Hind*III into an expression vector containing the alpha-myosin heavy chain promoter (gift from Dr. Jeffrey Robbins, University of Cincinnati, USA). At the C-terminus a HA-tag was fused by polymerase chain reaction (PCR) (S1 Table). Transgenic mice were generated by pronuclear microinjection according to standard procedures (Clara Christie Centre for Mouse Genomics, University of Calgary, Canada). Genomic DNA was prepared with the Extracta DNA Prep Kit (Quanta BioSciences, Gaithersburg, USA) from mice tails and founder mice were identified by PCR genotyping (S1 Table). Generation of transgenic mice and animal handling was performed in strict accordance with the recommendations in the Guide for the Care and Use of Laboratory Animals of the Canadian Council on Animal Care. The protocol was approved by the Animal Care Committee of the University of Calgary (Permit Number: AC14-0154*)*. All surgery was performed under isoflurane inhalation anesthesia with appropriate pain medication *(*Buprenorphine), and all efforts were made to minimize suffering.

### Echocardiographic analysis

Echocardiographic analysis of heart function was conducted using a Vevo 770 (Visual Sonics, Toronto, Canada) echocardiography system equipped with a 30 MHz transducer probe. The operator was blinded as to the genotype of mice. Mice were continuously anesthetized with 1.5–2% isoflurane (Pharmaceutical Partners of Canada, Richmond Hill, Canada) and warmed on a heated pad (37°C) for long and short axis views of heart function involving B-Mode and M-Mode measurements. Echocardiography was performed every two weeks, starting at five weeks of age and ending at 13 weeks of age. End systolic and diastolic diameters of the left chamber, interventricular septum and posterior wall thickness as well as left ventricular fractional shortening and ejection fraction were analyzed for each mouse in a blinded manner. All values were averaged by using five to ten cardiac cycles per mouse.

### Dissection of mouse hearts

Mice were anesthetized by using isoflurane and euthanized by cervical dislocation. Subsequently, hearts were dissected, washed with PBS and immediately frozen in liquid N_2_ and stored at -80°C.

### Histology and immunohistochemistry

Mice hearts were dissected, washed in phosphate buffered saline (PBS) and fixed in 4% paraformaldehyde overnight at 4°C. Subsequently, hearts were embedded in paraffin and sliced (5 μm) or cryoslides were prepared. Haematoxylin and eosin (HE), Masson trichrome (MTS), picro sirius red (PSR) and von Kossa (VK) staining was done as previously described (http://www.ihcworld.com). Slices were deparaffinized using xylene and ethanol, blocked with goat serum and stained over night with primary antibodies (S2 Table). After washing with PBS, slides were stained with secondary antibodies conjugated with Alexa-488 and Alexa-555 dyes for 2 h at room temperature (RT). Afterwards, samples were washed with PBS and embedded in proLong Gold antifade reagent containing 4’,6-Diamidin-2-phenylindol (DAPI, life technologies, Carlsbad, USA). Histology was analyzed with an Olympus BX54 microscope equipped with an UPlanSApo 100x/1.4NA objective (Olympus, Tokyo, Japan). The LSM5 Exciter (Carl Zeiss Microscopy, Oberkochen, Germany) was used for the confocal analysis. DAPI was excited at 405 nm and the emission was detected in a range between 420–480 nm. Alexa-488 was excited at 488 nm and the emission was detected between 505–530 nm. Alexa-555 was excited at 555 nm and the emission was detected between 560–615 nm. All images were processed with Zen software (Carl Zeiss Microscopy, Oberkochen, Germany).

### Western blot analysis

Frozen hearts were pulverized by pestle under liquid N_2_ and incubated with RIPA buffer supplemented with proteinase inhibitor cocktail (Roche, Mannheim, Germany) for 5 min at 4°C. The precellys-24 homogenizer (Bertin Technologies, France) was used to homogenize samples. Protein concentrations in the supernatants were determined with the Pierce BCA Protein Assay Kit (Thermo Scientific, Waltham, USA) and sodium dodecyl sulfate polyacrylamide gel electrophoresis (SDS-PAGE) was used to separate the proteins. Protein transfer and detection were carried out as previously described [26] by incubating with the primary antibodies over night at 4°C followed subsequently by incubation with HRP-conjugated secondary antibodies (S2 Table). Expression levels were normalized to loading controls. Subcellular protein fraction kit (#87790, Thermo Scientific) was used according to the manufacturer’s instruction to determine the percentage of membrane and cytoplasmic DSC2-HA. N-Cadherin and GAPDH were used as controls as membrane or cytoplasmic protein, respectively.

### Quantitative real time polymerase chain reaction (qRT-PCR)

Frozen hearts were pulverized under liquid N_2_. Afterwards, RNA was isolated with the PerfectaPure RNA fibrous tissue kit (5PRIME, Hilden, Germany) and concentrations were determined by using the NanoVue Plus spectrophotometer (GE Healthcare, Buckinghamshire, UK). QScript cDNA Supermix (Quanta BioSciences, Gaithersburg, USA) was employed to transcribe 200 ng RNA into cDNA. The Perfecta SYBR Green Super Mix was used in combination with appropriate primers (S1 Table) and a CFX96 Touch Real Time PCR system (BioRad, Hercules, USA) to determine relative expression levels. The mRNA expression levels were normalized relative to GAPDH mRNA by using the ΔΔC_t_ method [27]. Each qRT-PCR experiment was performed in triplicate.

### Telemetry

At ten weeks of age, ECG telemetry devices (Data Sciences International, St. Paul, USA) were implanted by ventral abdominal incision as previously described [28]. One week after surgery, electrocardiograms of free moving mice were recorded for 24 h using RPC-1 PhysioTel Receiver (Data Sciences International). Data were analyzed with LabChart V8.1 software (AD Instruments, Colorado Springs, USA).

### Microarray analysis

Microarray analysis was performed as previously described by using Mouse 1.0ST arrays (Affymetrix, Santa Clara, USA) [29]. Seven transgenic and seven control mouse hearts at the same age were compared for each time point. The data discussed in this publication have been deposited in NCBI's Gene Expression Omnibus and are accessible through GEO Series accession number GSE84645 (http://www.ncbi.nlm.nih.gov/geo/query/acc.cgi?acc=GSE84645).

The microarrays were analyzed in R/Bioconductor using the Robust Means Algorithm for normalization [30], followed by a limma package linear model fit and empirical Bayesian correction [31] (S1 File). Genes for which the log2-transformed control transgenic signal ratios were <-0.58 or >0.58 (±50% expression change) with a Benjamini-Hochberg adjusted false discovery rate of <0.05 were considered as genes whose expression were significantly changed by overexpressing DSC2. For pathway analyses the Database for Annotation, Visualization and Integrated Discovery 6.7 (DAVID, https://david.ncifcrf.gov) was used.

### Statistical analysis

Data of experimental groups were compared by non-parametric Mann-Whitney test using GraphPad Prism (Version 6, GraphPad Software, La Jolla, USA) and were presented as mean ± standard derivation (SD). Values of p≤0.05 were considered as significant.

## Results

### Stable transgenic DSC2 overexpression reveals patchy fibrosis in several mouse lines

**Fig 1:**
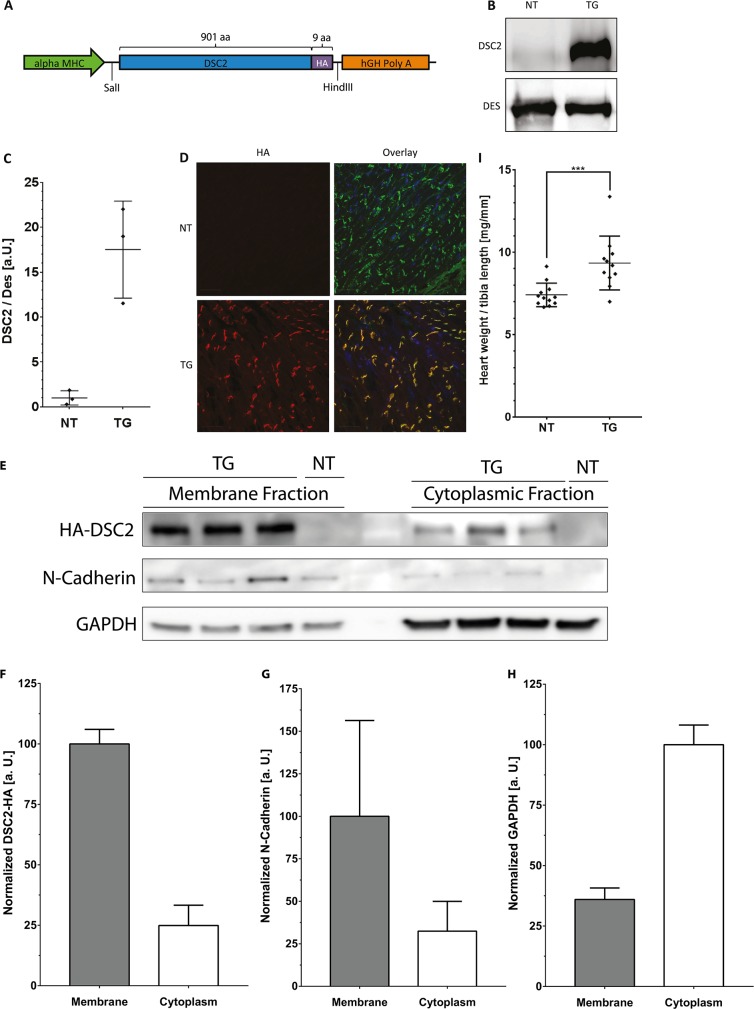
Generation and basic characterization of transgenic *DSC2* mice. **(A)** Design of the construct for generation of DSC2 transgenic mice. **(B)** DSC2 protein expression analysis. The intermediate filament protein DES (Desmin, 55 kDa) was used for loading control. **(C)** Quantification of DSC2 protein expression reveals a ~17-fold overexpression of DSC2 in comparison to endogenous Dsc2 by using an antibody recognizing endogenous murine Dsc2 and exogenous human DSC2 (95 kDa, Progen, Heidelberg, Germany). Data represent mean ± SD; n = 3; DES = desmin. **(D)** Immunohistochemistry of myocardial tissue using anti HA- (red) and N-Cadherin (green) antibodies. Scale bars represent 20 μm. Of note, exogenous DSC2 and N-Cadherin co-localize (yellow). **(E)** Membrane and cytoplasmic fractionation in combination with Western blot analysis for DSC2-HA and N-Cadherin and GAPDH as controls for membrane and cytoplasmic proteins. **(F-H)** Normalized quantification of membrane fraction of DSC2-HA **(F)**, N-Cadherin **(G)** and GAPDH **(H)**. **(I)** Heart weight / tibia length ratio of transgenic and non-transgenic mice (13 weeks), n = 12. Statistical analysis was performed by non-parametric Kruskal-Wallis test (***p<0.001).

We developed a transgenic mouse model with cardiac specific overexpression of DSC2 to examine if increased DSC2 expression induces a cardiac phenotype. An HA-tag was fused to the C-terminus of the human DSC2 cDNA (NM_024422.4) under the control of the cardiac specific myosin heavy chain promoter (Fig 1A). The construct was microinjected in oocyte pronuclei and several founder mice were identified (Figure A in S1 Dataset). Selected founder mice were outcrossed with C57BL6 wild-type animals and initial morphological analysis revealed severe cardiac fibrosis of offspring in four out of five generated lines (Figure Bin S1 Dataset). Next, the relative DSC2 expression level was analyzed by qRT-PCR and four out of five founder lines showed a relative expression between 0.70 ± 0.48 and 2.65 ± 0.44, whereas line 4 did not expressed exogenous DSC2 indicating a silenced chromosome integration (Figure C in S1 Dataset). The protein expression level of DSC2-HA in the different lines was further evaluated by Western blot analysis indicating similar results to the qRT-PCR analysis (Figure C in S1 Dataset). Because of moderate DSC2 expression levels mouse line 31 was selected for further analyses. The expression levels of endogenous and exogenous DSC2 were compared by using antibodies recognizing murine and human DSC2 indicating that exogenous DSC2 was about 17-fold higher expressed compared to endogenous Dsc2 (Fig 1B and 1C). IHC revealed that exogenous DSC2-HA was correctly processed and co-localized with N-Cadherin at the intercalated discs (Fig 1D).

To evaluate if the overexpressed DSC2-HA is properly localized at the intercalated discs we quantified the fractions of DSC2-HA between the cytoplasm and the membrane in transgenic and non-transgenic myocardial tissue, which revealed that the amount of DSC2-HA within the cytoplasm is about 25% of the amount of DSC2-HA at the intercalated discs (Fig 1E–1H) indicating that most of the overexpressed DSC2-HA is processed to the intercalated disc. As controls we also analysed the subcellular localization of N-Cadherin and GAPDH. As expected, these data showed for N-Cadherin (membrane protein) comparable results to DSC2-HA and for GAPDH (cytoplasmic protein) that the majority of the protein is localized in the cytoplasm.

Increased expression of glucose regulated protein 78 (GRP78) is a hallmark of ER stress which could be a non-specific effect of our transgene. Therefore, we performed Western blot analysis using GRP78 antibodies to proof if ER stress is involved in our transgenic mouse model. This experiment revealed a comparable expression level of GRP78 in transgenic and non-transgenic mice (Figure D in S1 Dataset).

Given our first evidence of visible patchy epicardial fibrosis, we measured heart weight / tibia length ratios of transgenic mice at 13 weeks of age (Fig 1I) which were significantly increased (NT vs. TG; 7.41 ± 0.72 vs. 9.34 ± 1.64 mg/mm; p<0.001). First characterization also included mRNA expression levels of common heart failure markers such as atrial and brain natriuretic peptides (ANP, BNP) showing a significant upregulation (NT vs. TG; ANP: 100 ± 68.6 vs. 241.3 ± 106.7 a. u.; p<0.001; BNP: 100 ± 58.4 vs. 519.9 ± 223.5 a. u.; p<0.0001; Figure E in S1 Dataset).

### Development of biventricular cardiomyopathy without arrhythmic events

**Fig 2:**
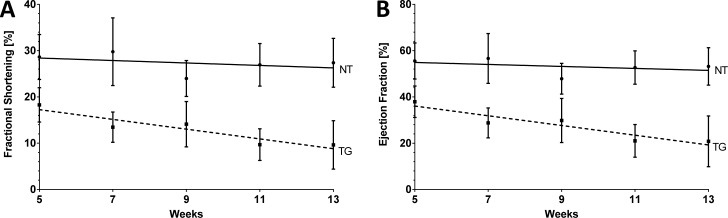
Functional cardiac assessment of transgenic *DSC2* mice using 2D echocardiography. Time dependent analysis of LV **(A)** fractional shortening and **(B)** ejection fraction of non-transgenic (solid line) and transgenic mice (dotted line). n(NT) = 12 (6 m, 6 f); n(WT) = 12 (6 m, 6 f). One DSC2 transgenic animal died at the age of 12 weeks. Data are presented as mean ± SD.

Transgenic mice were further assessed for cardiac function by serial 2D-echocardiography over a time frame of five to thirteen weeks. To exclude gender effects we investigated a mixed cohort consisting of six animals of both genders and corresponding control mice. Left ventricular fractional shortening (LV-FS) and ejection fraction (LV-EF) of transgenic hearts at five weeks of age were already significantly decreased (NT vs. TG; 28.6 ± 4.8 vs. 18.3 ± 3.7 a. u.; p<0.01) and deteriorated further by 13 weeks (NT vs. TG; 27.4 ± 5.3 vs. 9.6 ± 5.2 a. u.; p<0.0001; Fig 2 and Figure F in S1 Dataset). Of note, we could not detect any effect specific to gender (Figure G in S1 Dataset).

Left ventricular internal end-diastolic diameter (LVID_d_) was moderately increased in DSC2 transgenic animals at 13 weeks of age (NT vs. TG; 4.26 ± 0.23 vs. 4.82 ± 0.46 mm; p<0.01; Table 1) indicating left ventricular dilation. Septal and posterior wall thicknesses of the left ventricle (IVS_d_ and LVPW_d_) were only slightly changed between transgenic and control hearts excluding pathological hypertrophy (Table 1).

**Table 1:** Echocardiography values.

**Age [w]**		**Heart rate [bpm]**	**IVS**_**d**_ **[mm]**	**IVS**_**s**_ **[mm]**	**LVPW**_**d**_ **[mm]**	**LVPW**_**s**_ **[mm]**	**LVID**_**d**_ **[mm]**	**LVID**_**s**_ **[mm]**	**EF [%]**	**FS [%]**
5	NT (n = 12)	375.8 ± 55.1	0.73 ± 0.09	1.08 ± 0.12	0.86 ± 0.11	1.17 ± 0.14	4.25 ± 0.28	3.09 ± 0.36	55.16 ± 7.39	28.63 ± 4.85
	TG (n = 12)	379.8 ± 48.5	0.74 ± 0.14	0.92 ± 0.16	0.88 ± 0.22	1.16 ± 0.26	4.42 ± 0.47	3.57 ± 0.54	37.99 ± 6.82	18.29 ± 3.71
	P-value	n. s.	n. s.	0.0114	n. s.	n. s.	n. s.	n. s.	<0.0001	<0.0001
13	NT (n = 12)	379.5 ± 37.0	0.83 ± 0.16	1.21 ± 0.21	0.83 ± 0.15	1.20 ± 0.26	4.26 ± 0.23	3.10 ± 0.36	53.21 ± 8.12	27.80 ± 5.28
	TG (n = 11)	378.2 ± 23.5	0.68 ± 0.11	0.83 ± 0.15	0.83 ± 0.23	0.96 ± 0.28	4.82 ± 0.46	4.32 ± 0.50	20.79 ± 11.0	9.63 ± 5.24
	P-value	n.s.	<0.05	<0.0001	n. s.	<0.01	<0.01	<0.0001	<0.0001	<0.0001

Results are presented as mean ± standard deviation (SD). P-values based on Mann-Whitney test. IVS_d_ intra ventricular septum in diastole; IVS_s_ intra ventricular septum in systole; LVPW_d_ left ventricular posterior wall in diastole; LVPW_s_ left ventricular posterior wall in systole; LVID_d_ left ventricle inner diameter in diastole; LVID_s_ left ventricle inner diameter in systole; EF ejection fraction; FS fractional shortening.

In summary, the echocardiographic data confirm moderate left ventricular dilatation and significantly reduced cardiac function consistent with a phenotype of dilated cardiomyopathy.

Ventricular arrhythmias and sudden cardiac death are common signs of human cardiomyopathies. However, DSC2 transgenic mice did not suddenly die within their 13 weeks of lifetime. To detect potentially non-lethal arrhythmias in our mouse model *in vivo*, we implanted ECG transmitters and performed telemetry experiments over ten days under housing conditions. Severe frequent ventricular arrhythmias were not detected in free moving transgenic mice, however, ECG analyses showed prolonged QRS and QT_c_ intervals indicating prolonged ventricular conduction and depolarization (Figure H in S1 Dataset and S3 Table).

### Patchy fibrosis, necrosis and calcifications replacing the myocardium

**Fig 3:**
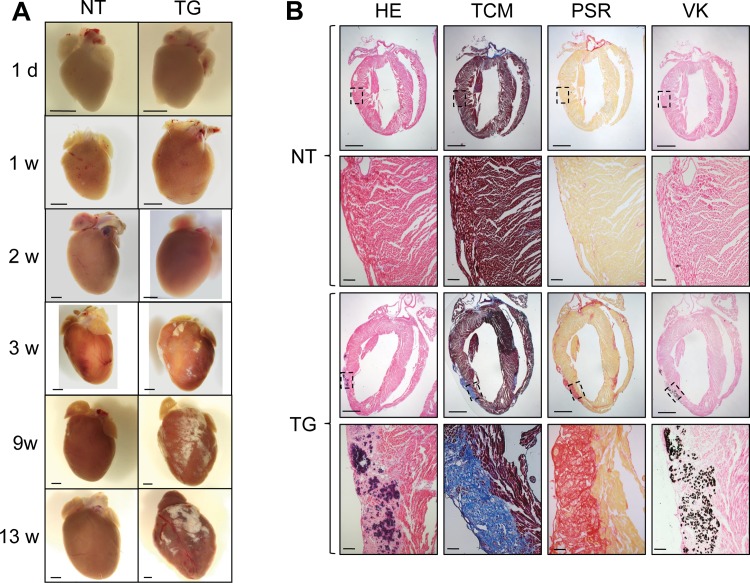
Structural analyses of transgenic mouse hearts. **(A)** Gross morphological analysis of transgenic and non-transgenic hearts (one day– 13 weeks). Of note, fibrosis was absent at day 1, week 1 and 2, but was present three weeks after birth. Scale bars represent 1 mm. **(B)** Histology of transverse sections of transgenic and non-transgenic hearts (9 weeks). HE = Haematoxilin and Eosin staining. TCM = Trichrome staining after Masson. PCR = Picro Sirius Red staining. VK = Von-Kossa staining. Scale bars represent 1 mm or 100 μm for the higher magnification. Of note, transgenic hearts demonstrate necrosis, extensive fibrosis and calcification mainly localized close to the epicardium.

Following our echocardiography analysis, we subsequently investigated in detail hearts of transgenic animals for structural changes over time by histology. Morphology and histology of dissected hearts of newborn and one week old transgenic mice appeared to be normal (Fig 3A and Figure I in S1 Dataset). However, first evidence for patchy fibrosis was seen between two and three weeks of age with increased severity over time (Fig 3A). Interestingly, fibrotic plaque areas were randomly distributed between hearts of different transgenic animals, but were commonly localized close to the epicardium and endocardium. Those plaques were seen in particular in the TCM staining and the PSR staining which highlights the extensive collagenase fibers in these locations (Fig 3B). HE-staining also revealed necrotic cardiomyocytes in addition to myocardial calcification observed in the VK staining (Fig 3B).

### Expression and localization of endogenous desmosomal proteins are changed locally

Overexpressing of one desmosomal component such as DSC2 might lead to alterations in the expression and localization of other desmosomal proteins. We used therefore qRT-PCR, Western blot analysis and IHC to analyze expression and protein localization of endogenous desmosomal proteins.

**Fig 4:**
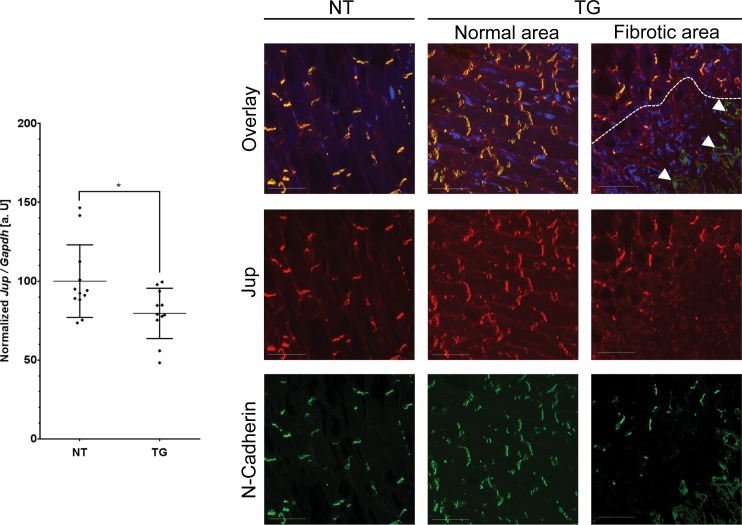
Structural and expression analysis of Jup using quantitative real-time polymerase chain reaction and immunohistochemistry. Expression analysis demonstrate a moderate but significant down-regulation mRNA of Jup (13 w; 12 NT; 11 TG). Statistical analysis was performed by non-parametric Kruskal-Wallis test (*p<0.05). Immunohistochemistry analysis of Jup (red) and N-cadherin (green) demonstrates the absence of Jup at the intercalated disc in fibrotic areas. The dashed line indicate the border between the fibrotic and normal areas. Of note, the cells within the fibrotic lesion are positive for N-Cadherin but negative for Jup (white arrows). The nuclei were stained with 4′,6-Diamidin-2-phenylindol (DAPI, blue). Scale bars represent 20 μm.

We observed significant down-regulation of endogenous mRNA and protein expression levels for Dsc2, Dsg2, Jup and Dsp in transgenic mice compared to non-transgenic mice (Fig 4 and Figures J-N in S1 Dataset). Only Pkp2 expression was not significantly changed at the mRNA and protein level (Figures L& N in S1 Dataset). When we examined in detail the localization of those endogenous desmosomal proteins at the intercalated discs, we found that they were almost absent in fibrotic areas but showed their normal localization in areas of remaining myocardium (Fig 4 and Figures J-Min S1 Dataset). This likely indicates a local remodeling process due to the replacement of cardiomyocytes, rather than a global downregulation of endogenous desmosomal genes.

### Gene expression pathway analyses reveal acute sterile inflammation, extracellular matrix remodeling and induction of fibrosis

Next, we performed serial microarray analyses to characterize gene expression changes which may contribute to the pathomechanisms in transgenic mice. Hearts of one and two week old animals did not show significant gene expression changes compared to their non-transgenic controls (data not shown), which is in good agreement with unremarkable histology observed at those early time points.

**Fig 5:**
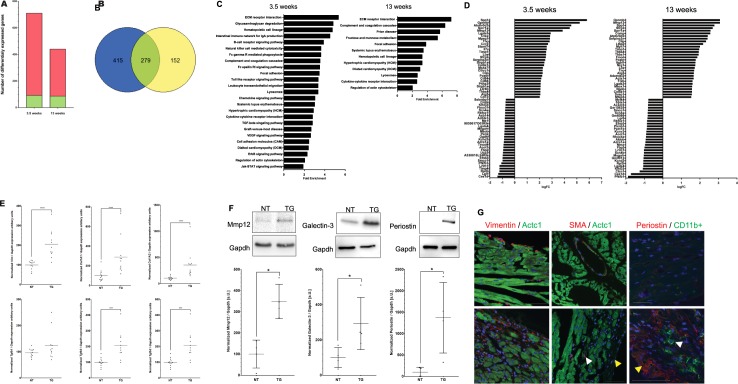
Gene expression analyses of DSC2 transgenic mouse hearts. **(A)** Diagram indicating the total number of differentially expressed genes (TG vs NT) identified by microarray analysis at different time points. p(adj)<0.05, logFC<-0.58 or >0.58. Red = upregulated genes, green = downregulated genes. **(B)** Venn diagram demonstrating the overlap (green) of differentially expressed genes between transgenic mice at the age of 3.5 weeks (blue) and 13 weeks (yellow). **(C)** KEGG analysis reveals activation of fibrotic and inflammatory pathways in hearts of transgenic mice. **(D)** Top30 up and down regulated genes in 3.5 and 13 week old transgenic animals.

In contrast, hearts from 3.5 weeks old transgenic animals demonstrated strong gene expression changes compared to their non-transgenic control hearts (Fig 5A). In brief, 91 genes were significantly down-regulated and 629 genes were up-regulated (p(adj)<0.05, logFC<-0.58 or >0.58). The expression of many genes involved in extracellular matrix (ECM) receptor interaction and cell adhesion were upregulated in 3.5 weeks transgenic mouse hearts, indicating activation of cardiac fibrosis and remodeling processes (Fig 5C). For instance, different structural ECM proteins like different collagens (e.g. *Col8a1*, logFC = 2.37; *Col12a1* logFC = 1.83; *Col1a2* logFC = 1.46) or fibronectin (*Fn1*, logFC = 1.77) were highly up-regulated in addition to genes encoding matricellular proteins like osteopontin (also known as secreted phosphoprotein-1, *Spp1*, logFC = 5.81), tenascin-C (*Tnc*, logFC = 2.81) or thrombospondin-1-4 (*Thbs1*, logFC = 1.76; *Thbs2*, logFC = 0.73; *Thbs3*, logFC = 0.67 *Thbs4*, logFC = 2.07). Additionally, members of the integrin receptor family connecting the ECM with the sarcolemma were up-regulated (*Itga4*, logFC = 0.96; *Itgb2*, logFC = 1.90; *Itgax*, logFC = 1.96). Moreover, genes encoding enzymes involved in ECM remodeling like matrix metalloproteinases (e.g *Mmp12*, logFC = 3.86), xylosyltransferase-1 (*Xylt1*, logFC = 0.59) or sulfatase-1 (*Sulf1*, logFC = 0.78) were up-regulated. Remarkably, tissue inhibitor of metalloproteinase-1 (*Timp1*, logFC = 2.53) was also highly increased, though Timp4 was significantly decreased (logFC = -1.22). Overall, those gene expression changes indicate ECM remodeling and induction of cardiac fibrosis.

Furthermore, several pathways involved in inflammatory response like cytokine-cytokine receptor interaction, chemokine signaling pathway or Toll-like receptor signaling were highly activated. For example, different chemokines (e.g. *Ccl3*, logFC = 1.90) and chemokine receptors (e.g. *Ccr2*, logFC = 2.68), toll-like receptors (e.g. *Tlr9*, logFC = 1.78) as well as different interleukines (e.g. *Il33*, logFC = 1.35; *Il6*, logFC = 0.84) and interleukin receptors (e.g. *Il7r*, logFC = 2.58) showed an increased expression. These results indicate acute sterile cardiac inflammation.

For end-stage transgenic animals at the age of 13 weeks we identified 85 down- and 352 upregulated genes (Fig 5A). 279 of these differently expressed genes were identified also in the 3.5 weeks old DSC2 transgenic animals (Fig 5B). However, acute inflammatory pathways, which were powerfully activated in younger transgenic animals, like chemokine signaling or toll-like receptor signaling were only slightly increased in older animals (Fig 5D).

**Fig 6:**
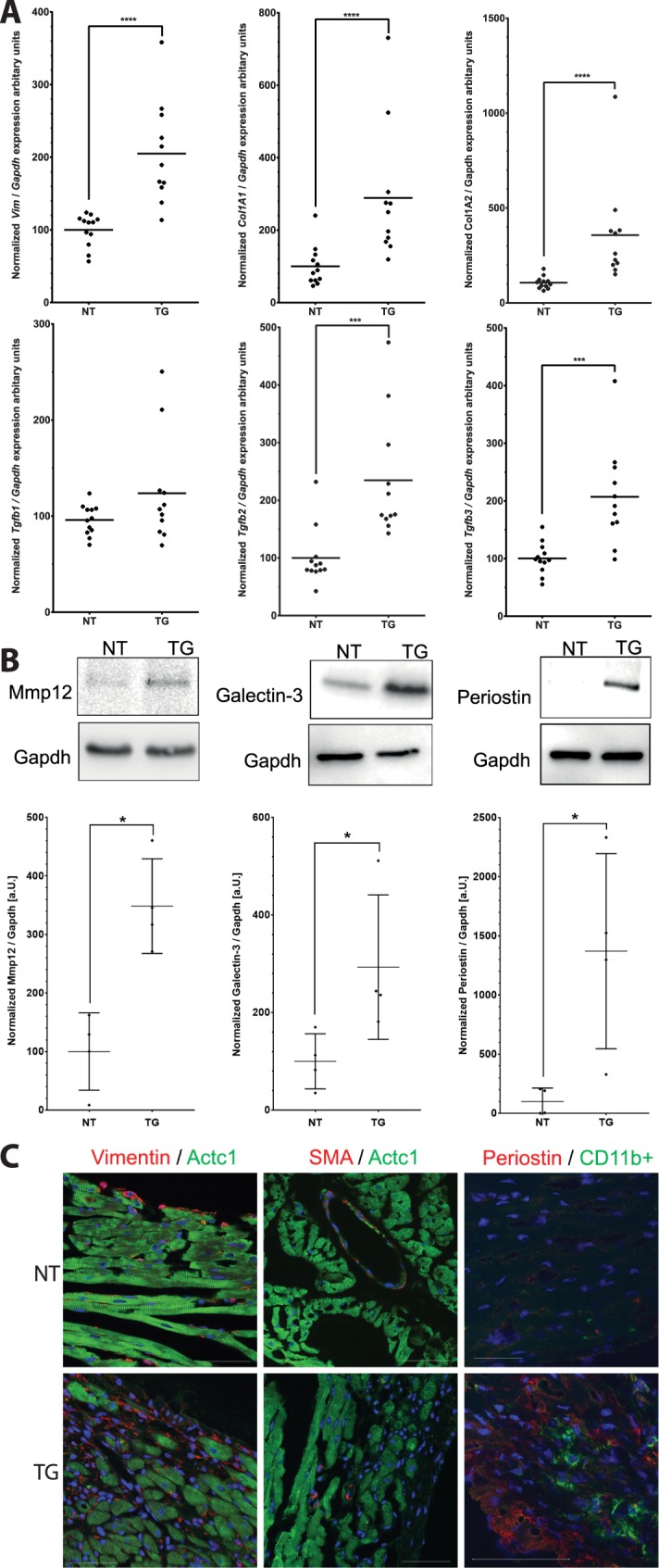
Confirmation of selected differentially expressed genes. Verification of selected differentially expressed genes n(TG) = 11 vs. n(NT) = 12 by quantitative real time PCR **(A)**. Statistical analysis was performed by non parametric Kruskal-Wallis test. *p<0.05; **p<0.01; ***p<0.001 and ****p<0.0001. **(B)** Western Blot analysis of Mmp-12, Galectin-3 and Periostin in n(TG) = 4 vs n(NT) = 4. Gapdh expression was used as a loading control. Statistical analysis was performed by non parametric Kruskal-Wallis test. *p<0.05. **(C)** Immunohistochemistry of Vimentin (red) / cardiac Actin (Actc1, green); alpha smooth muscle actin (SMA, red) / cardiac Actin (green) and Periostin (red) / CD11b+ (green). Vimentin indicates fibroblasts (white arrows). SMA is a marker of activated myofibroblasts (yellow arrow). Of note, many cells within the fibrotic area are SMA negative, but vimentin positive. CD11b+ is a molecular marker of macrophages (yellow arrow). Periostin is an important matricellular protein, which is highly expressed in fibrotic tissue. Scale bars represent 20 μm.

We selectively verified some differentially expressed genes like *Vim*, *Col1a1*, *Col1a2*, *Tgfb2*and *Tgfb3* by qRT-PCR (Fig 6A). The pro-fibrotic cytokine *Tgfb1* was not identified in the microarray analysis, however, we also examined *Tgfb1* mRNA expression changes, and observed an increased mRNA expression of *Tgfb2* and *Tgfb3* but not of *Tgfb1* in end-stage transgenic hearts was observed (Fig 6A). Furthermore, we performed Western Blot analyses for Mmp12, Galectin3 (*Lgals3*) and Periostin (*Postn*) and demonstrated that protein expression changes correspond to mRNA expression changes (Fig 6B).

To verify fibrotic and inflammatory remodeling, we performed IHC and investigated the expression of vimentin as a fibroblast marker, α-smooth muscle actin (SMA) as a marker for myofibroblasts and CD11b+ as a marker for macrophages (Fig 6C). Fibroblasts, myofibroblasts and also macrophages were more abundant in transgenic hearts, indicating inflammatory and fibrotic remodeling at the cellular level and suggesting a complex cellular and molecular cross-talk between cardiomyocytes, (myo)fibroblasts and macrophages contributing to the myocardial remodeling.

## Discussion

Desmosomal gene mutations have been shown to cause heart failure and arrhythmias mainly leading to AC or other primary cardiomyopathies [3, 5]. AC is often referred as a ‘*disease of the cardiac desmosomes*’, however the cellular and molecular events involved in the disease pathogenesis are complex and still poorly understood.

Various heterozygous [5], homozygous [32–34] or digenetic [35] missense or nonsense mutations have been described in *DSC2* in humans. It is still unclear, whether gain of function, loss of function or even both are relevant for the disease state and how the homeostasis of the cardiac desmosome might be influenced or even disturbed.

Several mouse models have been developed for Dsg2 [15, 20–22, 36], Jup [12, 23, 13, 37], Pkp2 [16, 24, 38], Dsp [17] and Des [39] showing either early embryonic lethality or different cardiac pathologies.

Surprisingly, the global and cardiac specific knock-out of Dsc2 is viable and did not develop a cardiac phenotype under housing conditions [25]. To our knowledge, no other genetic Dsc2 mouse model has been described. Therefore, we generated and characterized transgenic mice with cardiac specific overexpression of DSC2. Functional analyses revealed deteriorating cardiac function at 5 weeks of age, leading to more severe cardiac dysfunction and profound ventricular remodeling responses at 13 weeks of age. However, long term telemetry experiments excluded sustained ventricular arrhythmia, which is in agreement with absence of sudden cardiac death in those mice.

**Fig 7:**
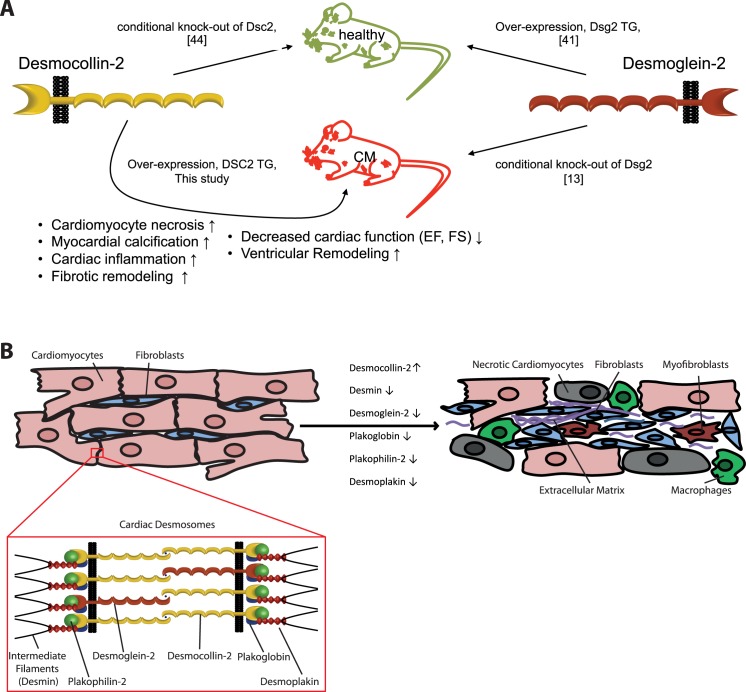
Schematic diagrams of mouse models and cellular remodeling processes targeting cardiac desmosomal proteins. **(A)** Cardiac specific knock-out of desmoglein-2 induces severe cardiomyopathy in mice [22]. Desmoglein-2 over-expressing mice are healthy and vital [20]. In contrast our study in combination with data from Rimpler [25] indicates a reverse situation for desmocollin-2. Transgenic mice, with a cardiac specific overexpression of desmocollin-2, develop cardiomyocyte necrosis, calcification, extensive fibrosis and scar tissue formation leading to significant decreased ejection fraction (EF) and fractional shortening (FS) indicating reduced systolic heart function. The underlying structural remodeling processes were associated with activation of different pro-fibrotic and pro-inflammatory cellular and molecular pathways. **(B)** Schematic overview about cellular remodeling caused by knock-out (↓) or overexpression (↑) of important cardiac desmosomal proteins. The remodeling process includes necrosis of the cardiomyocytes, proliferation of (myo)fibroblasts, expression of collagens and other extracellular matrix proteins and infiltration by macrophages.

Remarkably, cardiac phenotypes in mice for the desmosomal cadherin Dsg2, the counterpart of Dsc2, are contrary (Fig 7A). The global knock-out of Dsg*2* is embryonically lethal [15] whereas the cardiac specific knock-out of Dsg2 causes severe cardiomyopathy [22]. In contrast, transgenic mice overexpressing cardiac Dsg2 are without an obvious phenotype [20]. We present data supporting that for the counterpart Dsc2 the situation appears to be the reverse.

The desmosomal cadherins Dsc2 and Dsg2 are involved in direct cardiomyocyte adhesion [10]. However, cis- and trans-binding as well as homo- and heterophilic binding of both desmosomal cadherins are not completely understood at the molecular level and remain controversial. Garrod and co-workers speculated that desmosomes have a Ca^2+^-dependent adhesive state presumably associated with trans-binding of the first extracellular domains (EC1 and EC1*) and additionally form a Ca^2+^-independent hyper-adhesive state presumably stabilized by additional cis-binding of a β-helix within EC1 and EC2-EC3* [40, 41]. The contrary phenotypes of the knock-out and overexpressing mice for Dsg2 and Dsc2 suggest that both desmosomal cadherins may have different cis- and trans-binding functions. However, detailed biochemical binding studies (e.g. using scanning force microscopy) will be required to analyze specific structural and functional differences between desmosomal cadherins *in vitro* and *in vivo*.

Additionally, cardiac specific over-expression of adhesion proteins such as N-cadherin, E-cadherins or the Coxsackie-Adenovirus-receptor (CAR) also cause heart failure in mice [42, 43], suggesting that cardiomyocyte adhesion is sensitively controlled, dosage dependent, and needs fine-tuning and adjustments by different adhesion proteins.

Our DSC2 transgenic mice developed severe myocardial necrosis, fibrosis and calcification of both ventricles and the septum. Of note, the fibrotic plaque areas were randomly distributed for each transgenic mouse heart, but mainly localized close to the epicardium and sometimes endocardium. This has been also observed in human AC where the fibro-fatty infiltration process starts primarily at the epicardium.

Increasing fibrotic plaque areas are replacing the myocardium with intact desmosomal structures over time, which leads to extensive myocardial remodeling finally resulting in decreased cardiac function and a phenotype of progressive cardiomyopathy. As the remodeling process moves forward very quickly (between 2 and 3.5 weeks of age) the extent of ventricular dilation appears to be only moderate with mild changes in wall thicknesses. It is still unclear in human disease if structural derangements such as the fibro-fatty replacement are primary pathological events, or part of a secondary healing process caused by inflammatory injury and/or other cell signaling events [44–46]. Even if not directly comparable with human disease, our DSC2 mouse model shows interesting phenotypic features and gene expression changes mimicking a potential disease process seen in human cardiomyopathies and even other acute injuries of the heart. Although transgenic hearts look still unremarkable at two weeks of age, between two and three weeks we observed a dramatic switch where transgenic hearts quickly developed severe fibrotic patches with profound changes in gene expression, macroscopic and microscopic tissue changes suggesting an acute process of molecular and cellular events leading to necrosis and inflammation within a very short time period during postnatal growth. At the age of 3.5 weeks upregulation of many differentially expressed genes involved in inflammatory response such as chemokine, cytokine or toll-like receptor signaling takes place (Fig 5C and 5D), which was further supported by IHC demonstrating macrophage infiltration into the myocardium (Fig 6C) indicating an acute cellular response of the innate immune system.

Cardiac fibrosis is associated with a complex remodeling process of the ECM. We observed that genes encoding for structural ECM proteins like collagens or fibronectin were strongly upregulated. Furthermore, enzymes involved in degradation or reorganization of the ECM were dramatically changed, e.g. different matrix metalloproteinases and members of the ADAM family involved in ECM degradation. Proteoglycans and heparan sulfate proteoglycans are also important compounds of the ECM. Interestingly, gene expression of enzymes involved in the proteoglycan biosynthesis like xylosyltransferase-1 (*Xylt1*) [47] or modifying heparan sulfate proteogylcans like sulfatase-1 (*Sulf1*) [48] was also increased contributing to ECM remodeling in transgenic hearts.

Additionally, important matricellular proteins like osteopontin, periostin, tenascin and thrombospondins showed profound changes in expression. Matricellular proteins bind to different cell receptors and have diverse biological functions. In combination with cytokines and chemokines matricellular proteins regulate cell-mediated immune response, wound healing and tissue repair after injury [49–51]. The observed gene expression changes of different cytokines, chemokines, growth factors and matricellular proteins suggest complex biochemical intercellular signaling leading to immune cell infiltration and fibrotic replacement of cardiomyocytes in transgenic mice. Our data support that AC is a complex cardiac disease involving several different cell types and different inter and intra-cellular signaling pathways. However, the first molecular event initially triggering this process remains to be the subject of further studies.

In summary, overexpression of DSC2 in mice leads to the development of severe cardiomyopathy, whereas the gene knock-out in mice does not [25] (Fig 7A). Functional, structural and expression analyses of the DSC2 transgenic mice revealed cardiomyocyte necrosis, an acute inflammatory process, ECM remodeling and fibrotic replacement leading to decreased cardiac function and cardiomyopathy. Our study supports the hypothesis that cardiomyocyte adhesion mediated by desmosomal proteins is a sensitive biological process. Changes of the homeostasis between desmosomal cadherins induce a cascade of different cell-cell interactions mediated by a complex network of various extracellular signaling molecules leading to profound cardiac remodeling (Fig 7B).

## Supporting information

S1 Dataset Supporting figures A-N.
(PDF)

S1 File Bioconductor analysis script. (DOCX)

S1 Table Overview about oligonucleotides. (DOCX)

S2 Table Overview about antibodies. (DOCX)

S3 Table Electrocardiogram values. (DOCX)
