# Supplementary material for: Transgenic mice overexpressing desmocollin-2 (DSC2) develop cardiomyopathy associated with myocardial inflammation and fibrotic remodeling
Source: PLoS One. 2017 Mar 24;12(3):e0174019. doi: 10.1371/journal.pone.0174019 (PMC5365111; doi:10.1371/journal.pone.0174019)
Supplement: S1 Dataset — Supporting figures A-N. (PDF) [file pone.0174019.s001.pdf]

S1 Dataset

Figures A-N

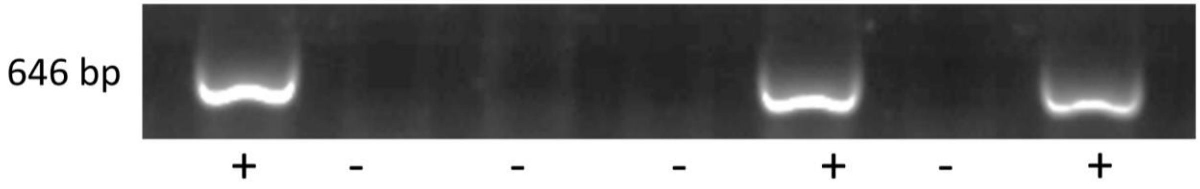

Figure A: DNA agarose gel of genotyping PCRs of transgenic founder mice.

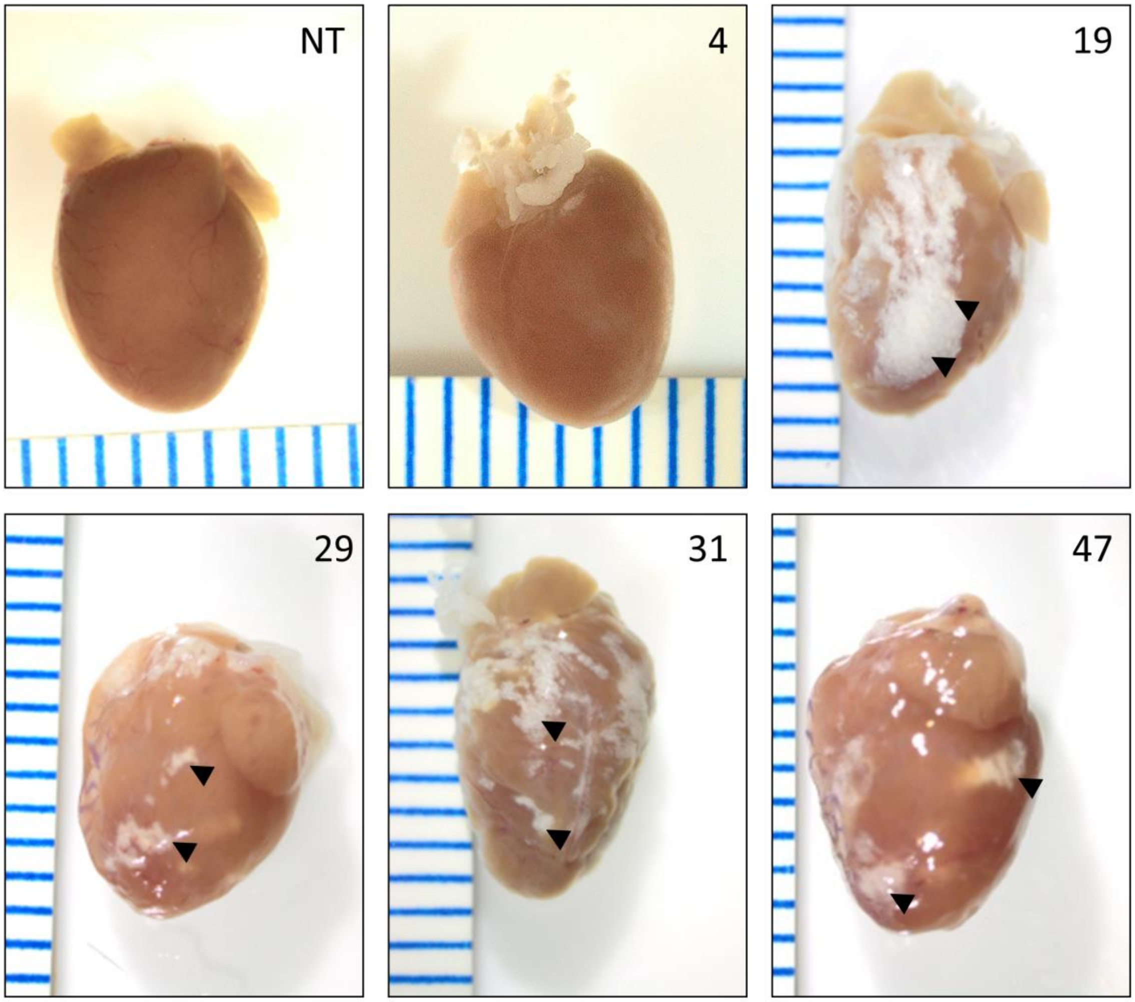

Figure B: Morphological analysis of dissected hearts (9 weeks) from a non-transgenic mouse and from different founder lines (4; 19; 29; 31; 47). Of note, fibrous areas (black arrows) were present in four of the founder lines (19; 29; 31; 47). Scale bars represent 1 mm.

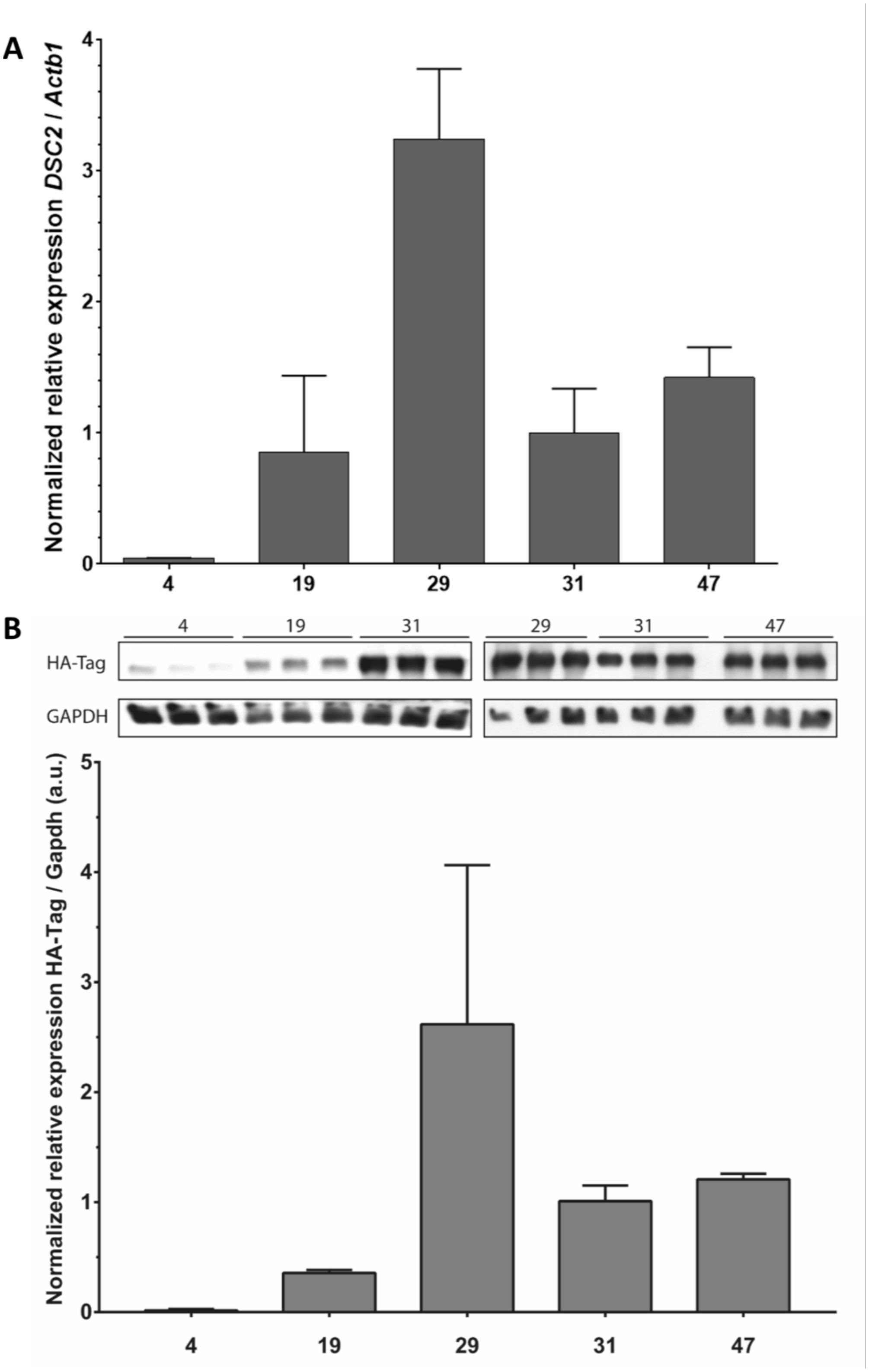

Figure C: DSC2 expression analysis using qRT-PCR **(A)** and Western blot analysis using HA-tag antibodies **(B)**. Data represent mean ± SD.

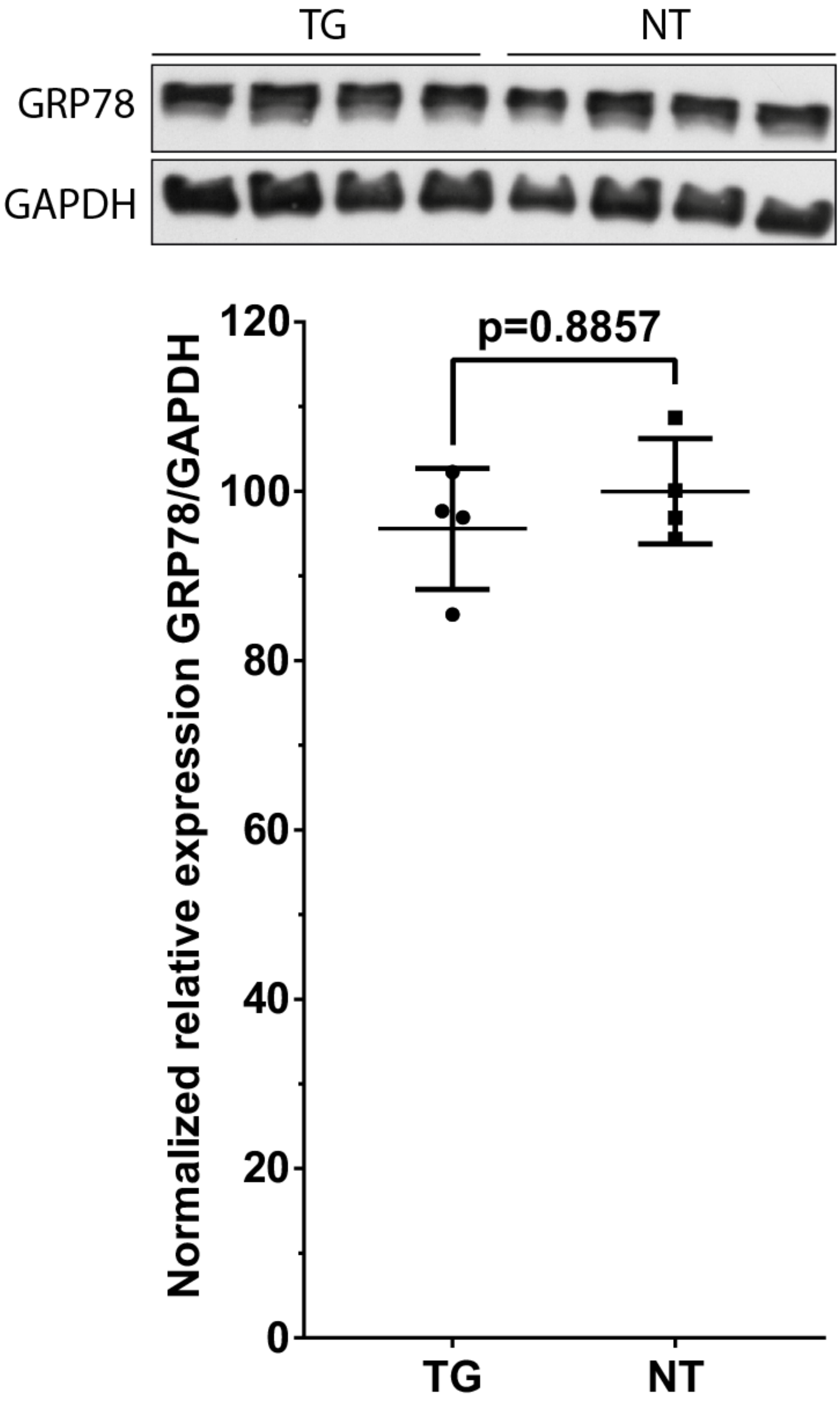

Figure D: Myocardial GRP78 protein expression in transgenic (TG) and non transgenic mice. Data represent mean ± SD.

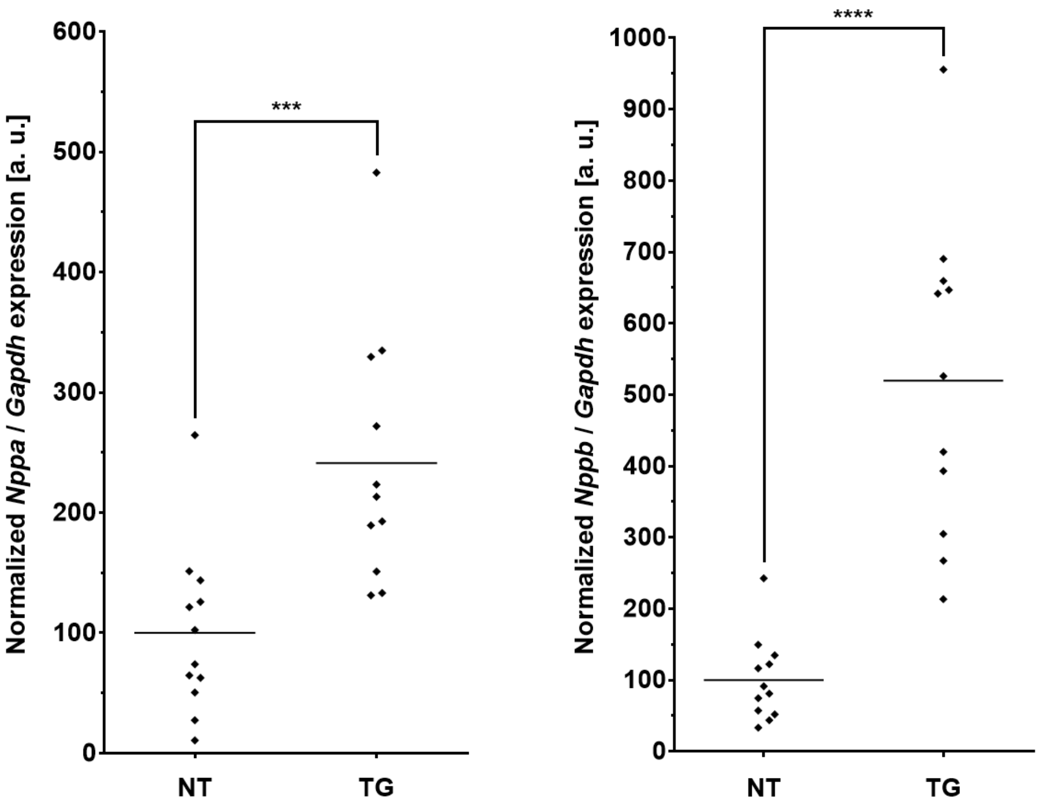

Figure E: *Nppa* (ANP) and *Nppb* (BNP) mRNA myocardial expression in transgenic and non-transgenic mice (13 weeks)**.** Data represent mean ± SD.

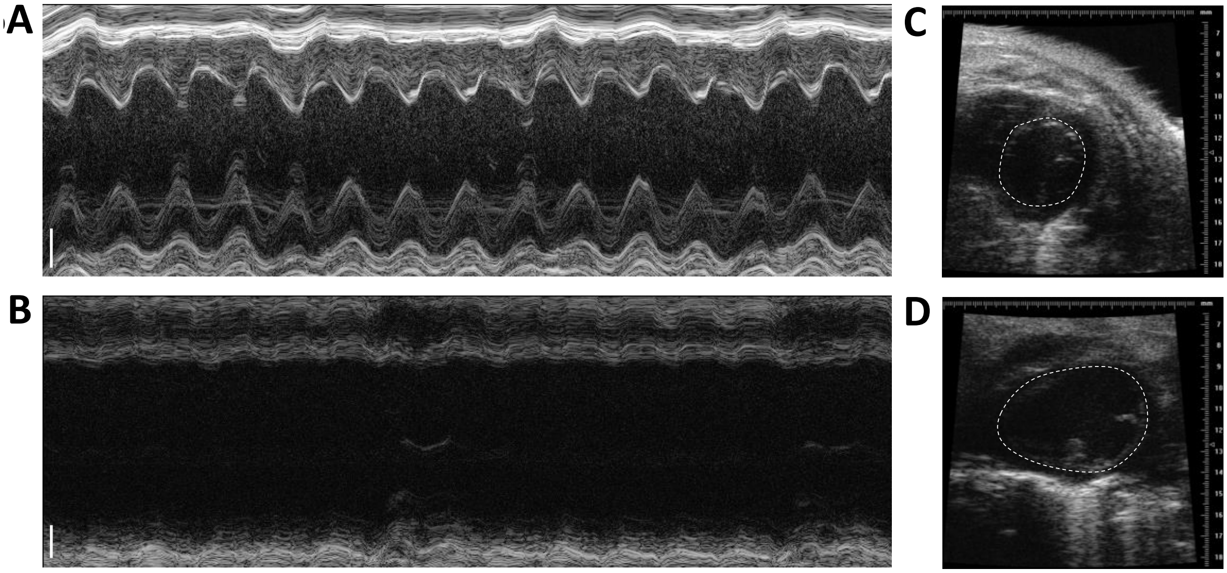

Figure F: Representative images of M-mode **(A-B)** and B-mode **(C-D)** echocardiography heart analysis of non-transgenic **(A, C)** and DSC2 transgenic animals **(B, D)**.

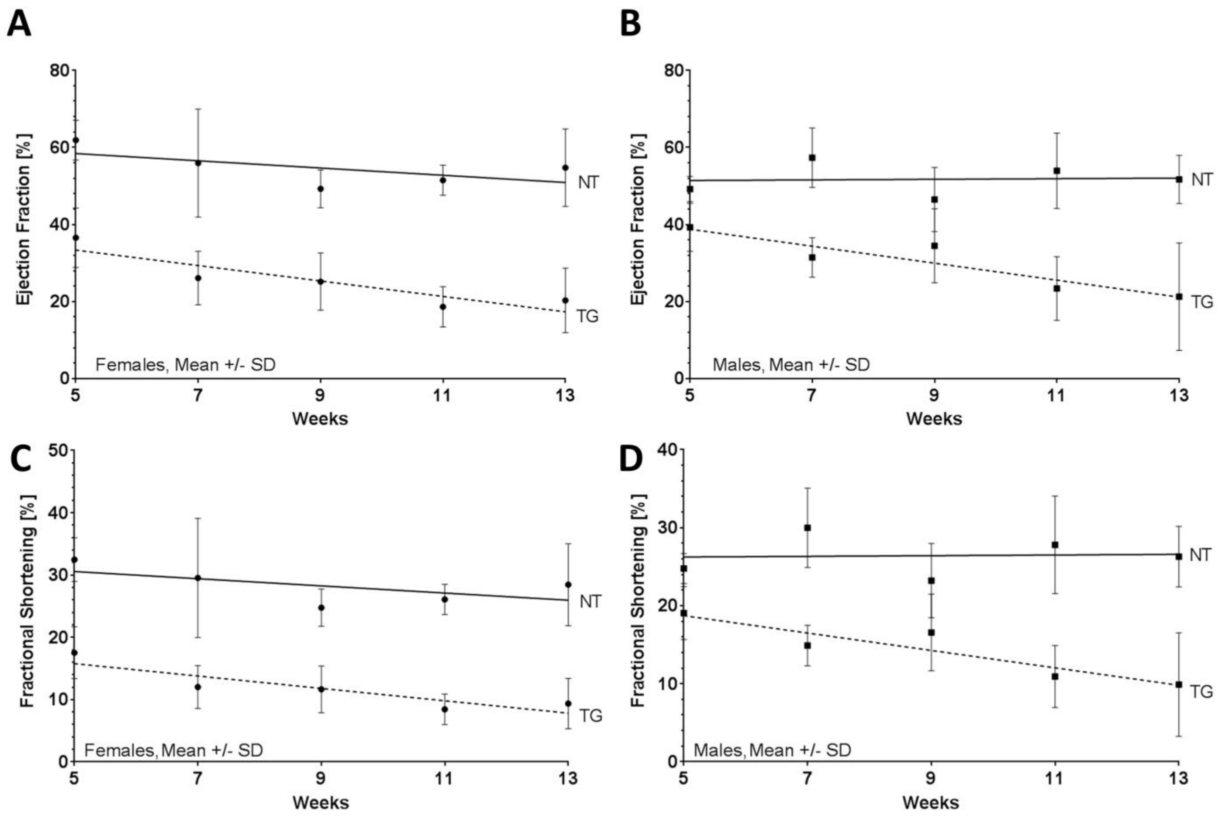

Figure G: Time dependent analysis of **(A-B)** ejection fraction and **(C-D)** fractional shortening of females **(A-C)** and males **(B-D)** between 5-13 weeks. The solid lines represent data of non-transgenic animals and the dotted line represents data of the transgenic animals. Data are presented as mean ± SD. One DSC2 transgenic male died at the age of 12 weeks.

F

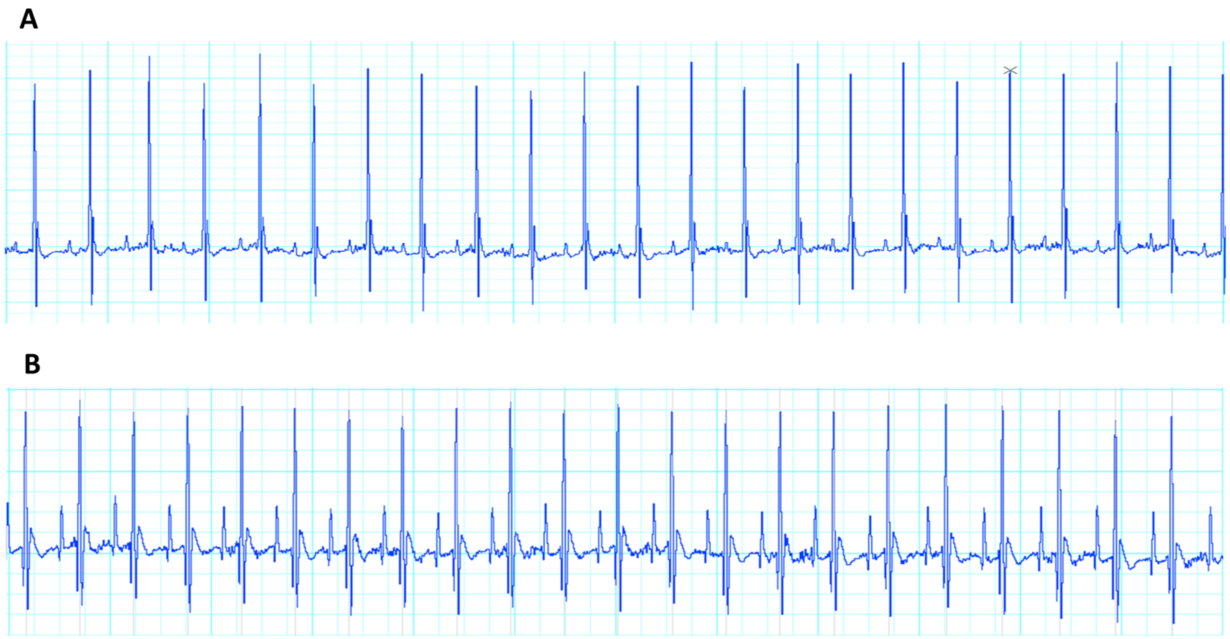

Figure H: Representative electrophysiological analysis of non-transgenic control mouse **(A)** and a transgenic DSC2 mouse **(B)** by using telemetry.

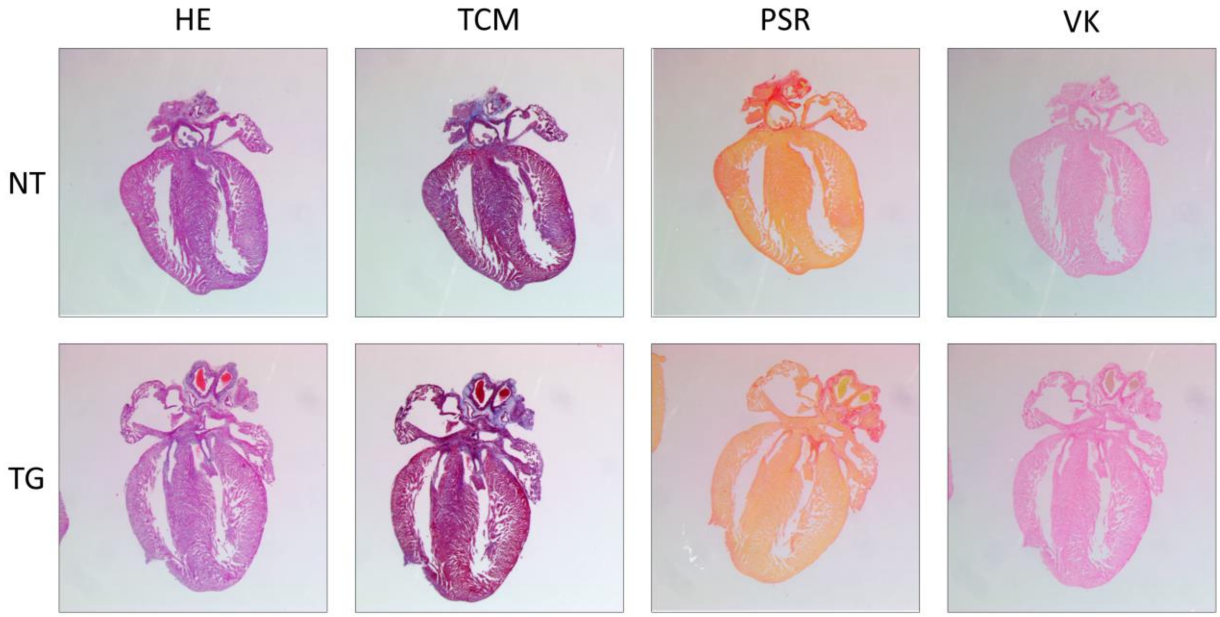

Figure I: Histology non-transgenic and transgenic new born animals. HE = Haematoxilin and Eosin staining. TCM = Trichrome staining after Masson. PCR = Picro Sirius Red staining. VK = Von-Kossa staining.

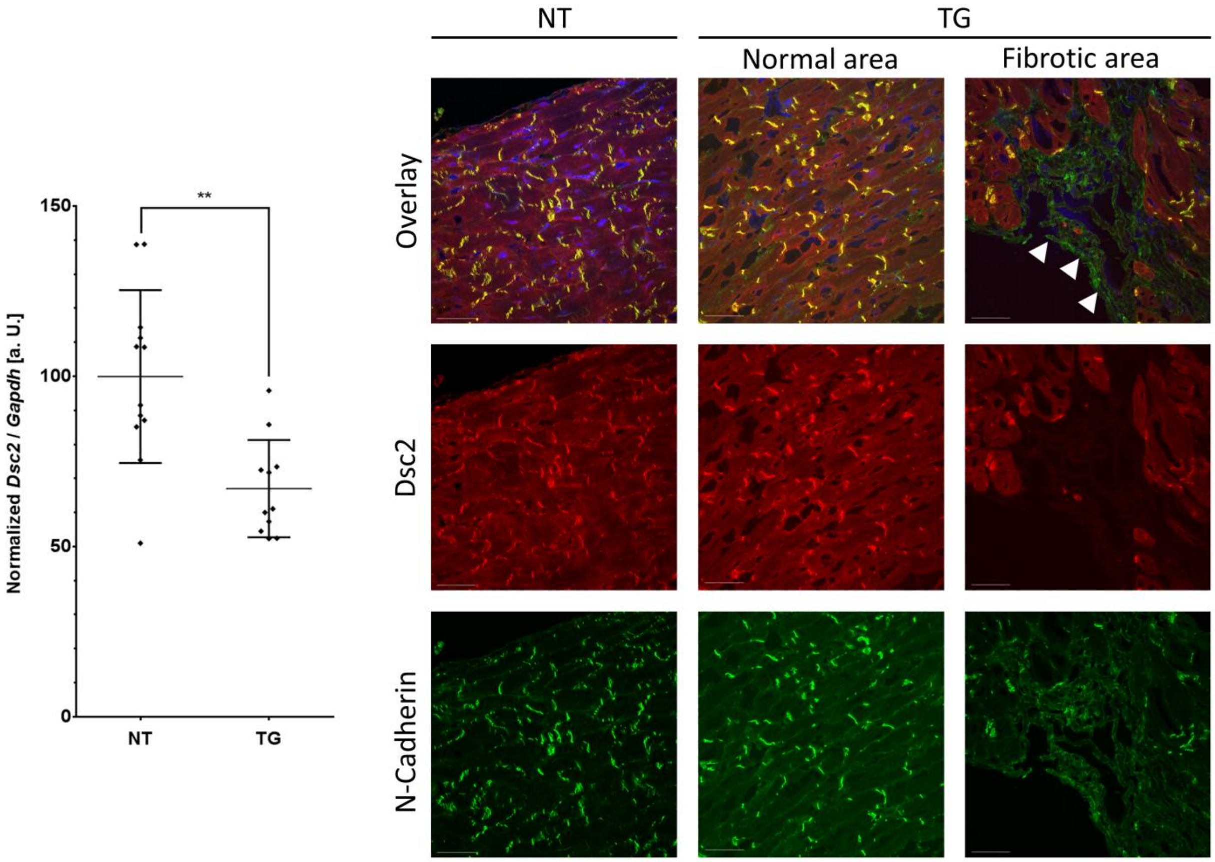

Figure J: Structural and expression analysis of Dsc2 using quantitative real-time polymerase chain reaction and immunohistochemistry. Normalized mRNA expression of endogenous Dsc2 (13 w; 12 NT; 11 TG). Statistical analysis was performed by non-parametric Kruskal-Wallis test (**$p<0.01$). Immunohistochemistry analysis of Dsc2 (red) and N-cadherin (green). Of note, the cells within the fibrotic area are positive for N-Cadherin but negative for Dsc2 (white arrows). The nuclei were stained with 4′,6-Diamidin-2-phenylindol (DAPI, blue). Scale bars represent 50 µm.

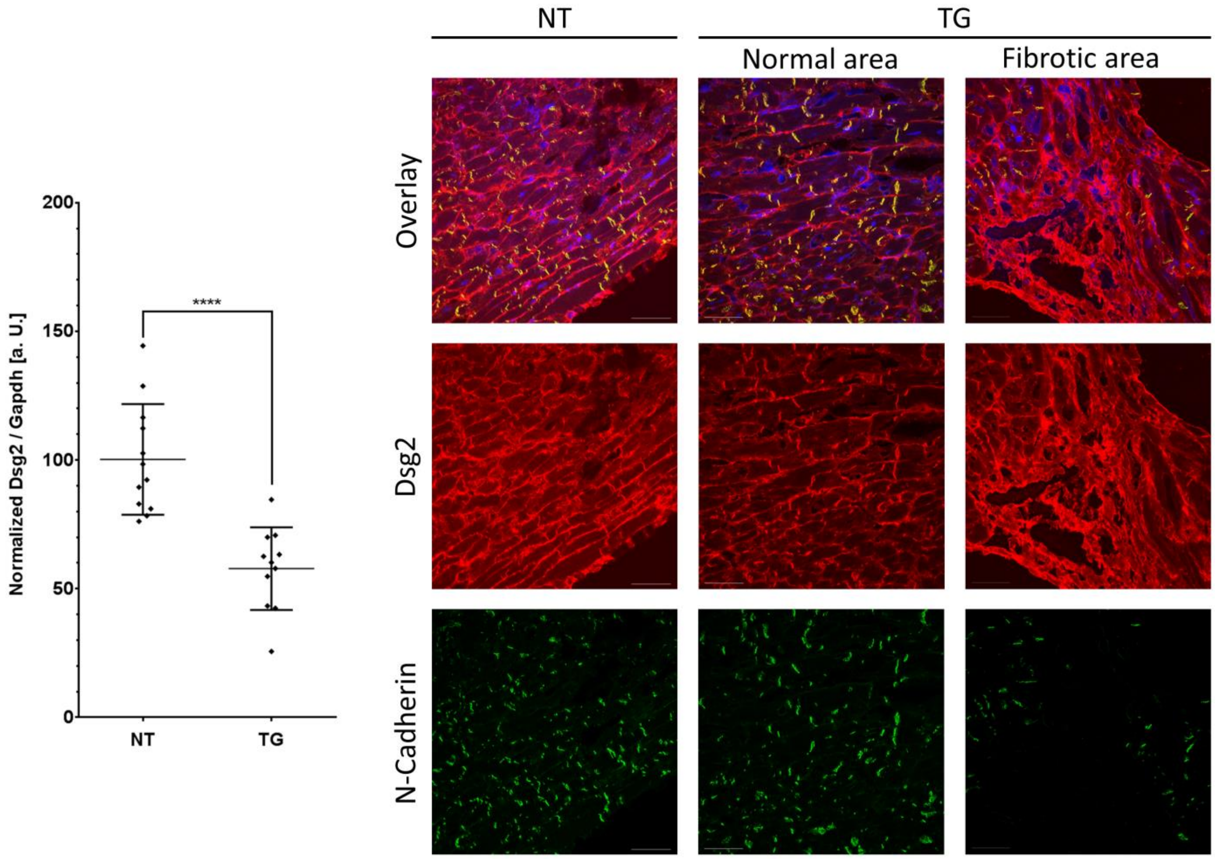

Figure K: Structural and expression analysis of Dsg2 using quantitative real-time polymerase chain reaction and immunohistochemistry. Normalized mRNA expression of endogenous Dsg2 (13 w; 12 NT; 11 TG). Statistical analysis was performed by non-parametric Kruskal-Wallis test (****p<0.0001). Immunohistochemistry analysis of Dsg2 (red) and N-cadherin (green). The nuclei were stained with 4′,6-Diamidin-2-phenylindol (DAPI, blue). Scale bars represent 50 µm.

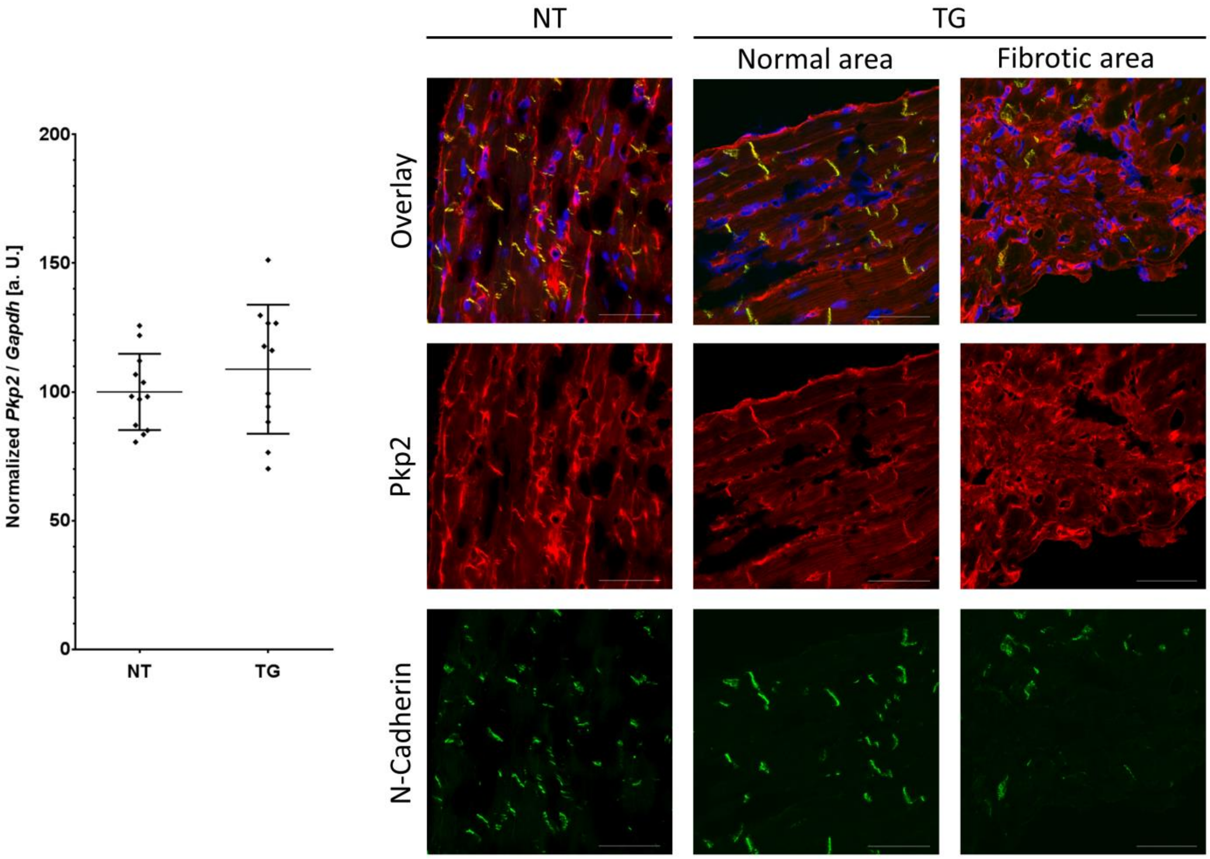

Figure L: Structural and expression analysis of Pkp2 using quantitative real-time polymerase chain reaction and immunohistochemistry. Normalized mRNA expression of endogenous Pkp2 (13 w; 12 NT; 11 TG). Statistical analysis was performed by non-parametric Kruskal-Wallis test (no significant differences). Immunohistochemistry analysis of Pkp2 (red) and N-cadherin (green). The nuclei were stained with 4′,6-Diamidin-2-phenylindol (DAPI, blue). Scale bars represent 50 µm.

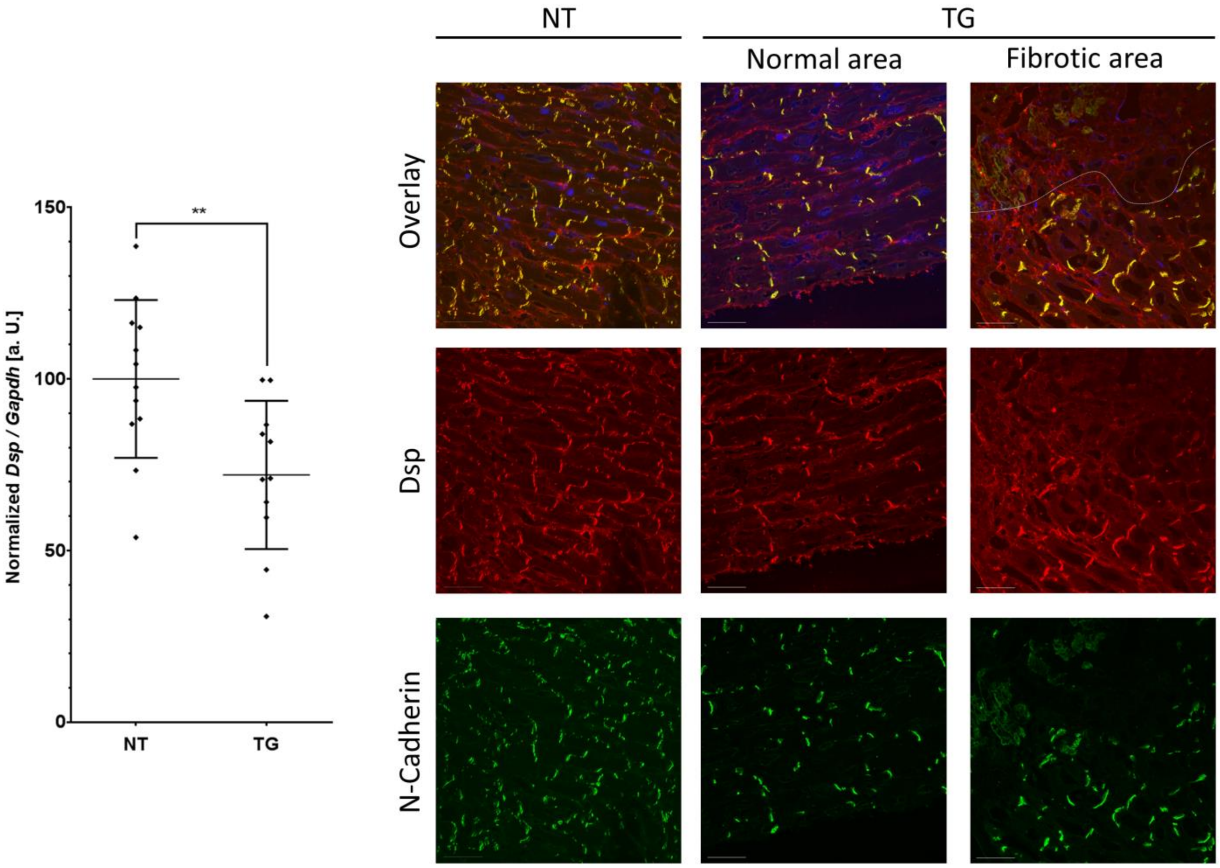

Figure M: Structural and expression analysis of Dsp using quantitative real-time polymerase chain reaction and immunohistochemistry. Normalized mRNA expression of endogenous Dsp (13 w; 12 NT; 11 TG). Statistical analysis was performed by non-parametric Kruskal-Wallis test (**$p<0.01$). Immunohistochemistry analysis of Dsp (red) and N-cadherin (green). The dashed line indicates the border between the fibrotic and normal areas. The nuclei were stained with 4′,6-Diamidin-2-phenylindol (DAPI, blue). Scale bars represent 50 µm.

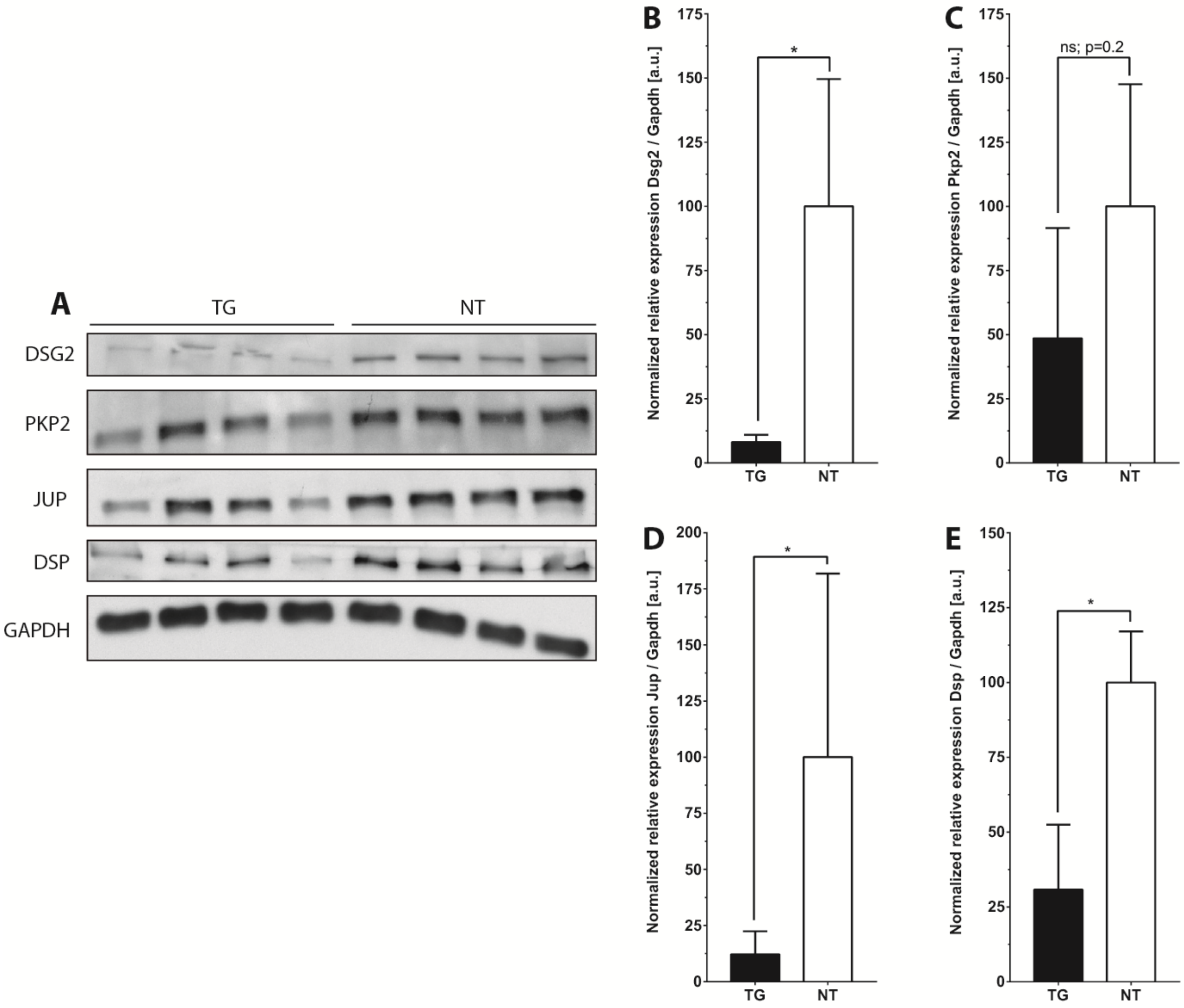

Figure N: Myocardial expression analysis of endogenous desmosomal proteins in DSC2-transgenic (TG) and non-transgenic animals using Western blot analysis **(A)**. Normalized relative protein expression of endogenous Dsg2 **(B)**, Pkp2 **(C)**, Jup **(D)** and Dsp **(E)** in DSC2-transgenic and non-transgenic animals (N=4). Statistical analysis was performed by non-parametric Kruskal-Wallis test; *$p<0.05$.
