# Supplementary material for: Transgenic mice overexpressing desmocollin-2 (DSC2) develop cardiomyopathy associated with myocardial inflammation and fibrotic remodeling
Source: PLoS One. 2017 Mar 24;12(3):e0174019. doi: 10.1371/journal.pone.0174019 (PMC5365111; doi:10.1371/journal.pone.0174019)
Supplement: S1 File — (DOCX) [file pone.0174019.s002.docx]

**S1 Bioconductor Analysis Script**

library(affy)

library(limma)

library(mogene10sttranscriptcluster.db)

phenoData <- read.AnnotatedDataFrame("covdesc")

eset <- justRMA(phenoData=phenoData)

gns <- select(mogene10sttranscriptcluster.db, featureNames(eset),

c("ENSEMBL","SYMBOL","GENENAME"))

gns <- gns[!duplicated(gns[,1]),]

eset_3w <- eset[,eset$age == "3w"]

eset_13w <- eset[,eset$age == "13w"]

eset_13w$treatment <- factor(eset_13w$treatment)

design_3w <- model.matrix(~ treatment, pData(eset_3w))

design_13w <- model.matrix(~ treatment, pData(eset_13w))

fit_3w <- lmFit(eset_3w, design_3w)

fit_13w <- lmFit(eset_13w, design_13w)

efit_3w <- eBayes(fit_3w)

efit_13w <- eBayes(fit_13w)

efit_3w$genes <- gns

efit_13w$genes <- gns

sig_13w <- topTable(efit_13w, coef=2, p.value=0.05, number=100000)

sig_3w <- topTable(efit_3w, coef=2, p.value=0.05, number=100000)

write.csv(sig_3w, file="3w.csv")

write.csv(sig_13w, file="13w.csv")
