# Supplementary material for: Transgenic mice overexpressing desmocollin-2 (DSC2) develop cardiomyopathy associated with myocardial inflammation and fibrotic remodeling
Source: PLoS One. 2017 Mar 24;12(3):e0174019. doi: 10.1371/journal.pone.0174019 (PMC5365111; doi:10.1371/journal.pone.0174019)
Supplement: S1 Table — (DOCX) [file pone.0174019.s003.docx]

| Oligonucleotide | Gene | NO. | Sequence [5’-3’] | Application |
| --- | --- | --- | --- | --- |
| mGapdh-for | *Gapdh* | 153230 | AGGTTGTCTCCTGCGACTTCA | qRT-PCR |
| mGapdh-rev | *Gapdh* | 153231 | CCAGGAAATGAGCTTGACAAAGTT | qRT-PCR |
| mDsc2-for | *Dsc2* | 153224 | ATGCAGATGGGAGAAGCTGT | qRT-PCR |
| mDsc2-rev | *Dsc2* | 153225 | TGCAACAATTTCAGCAGAGG | qRT-PCR |
| mJup-for | *Jup* | 147564 | ATCCCGGCCATTGTGAAACTGC | qRT-PCR |
| mJup-rev | *Jup* | 147565 | AGGCCAATAGTTGCCTTGACCAG | qRT-PCR |
| mDsp-for | *Dsp* | 147600 | AGAGCCATGACTATTGCCAAGCTG | qRT-PCR |
| mDsp-rev | *Dsp* | 147601 | TGGAGCTCAAGGTCTTCGATGG | qRT-PCR |
| mDsg2-for | *Dsg2* | 153226 | GCGCGTACTCCTCTAACACC | qRT-PCR |
| mDsg2-rev | *Dsg2* | 153227 | ACCTTCTGACTCTGCCTGGA | qRT-PCR |
| mPkp2-for | *Pkp2* | 153234 | GGCTCTCCAGAACCTCACAG | qRT-PCR |
| mPkp2-rev | *Pkp2* | 153235 | GGGAAAGATTCCGTGACAAA | qRT-PCR |
| mNppa-for | *Nppa* | 147594 | ATCCTGTGTACAGTGCGGTGTC | qRT-PCR |
| mNppa-rev | *Nppa* | 147595 | TCCAGGTGGTCTAGCAGGTTCTTG | qRT-PCR |
| mNppb-for | *Nppb* | 147596 | TCAGTCGTTTGGGCTGTAACGC | qRT-PCR |
| mNppb-rev | *Nppb* | 147597 | ACTTCAAAGGTGGTCCCAGAGC | qRT-PCR |
| DSC2_Ex2/3-for | *DSC2* | 141476 | CTTGTTGGTAGAGTTAACCTGAAAGAGTGC | Genotyping |
| DSC2_Ex6/7-rev | *DSC2* | 141477 | CCACAGTAGTGCCCACTCTGC | Genotyping |
| SalI_DSC2_for | *DSC2* | 133599 | GCGGCCGCGTCGACATGGAGGCAGCCCGCCC | Cloning |
| HindIII_HA_DSC2_rev | *DSC2* | 133601 | GGTACCAAGCTTAGCGTAATCTGGAACATCGTATGGGTATCTCTTCATGCATGCTTCTGCTAGTGTCCTAAATTTGG | Cloning |

**Table S1: Overview about oligonucleotides.**

Restriction sites are solid underlined and the HA-tag encoding sequence is dotted underlined.
