# Supplementary material for: Transgenic mice overexpressing desmocollin-2 (DSC2) develop cardiomyopathy associated with myocardial inflammation and fibrotic remodeling
Source: PLoS One. 2017 Mar 24;12(3):e0174019. doi: 10.1371/journal.pone.0174019 (PMC5365111; doi:10.1371/journal.pone.0174019)
Supplement: S2 Table — (DOCX) [file pone.0174019.s004.docx]

**Table S2. Overview about the used antibodies.**

| Antibody | Company | Number | Application | Dilution |
| --- | --- | --- | --- | --- |
| MS anti-N-Cadherin | BD Transduction Laboratories | 610920 | IHC | 1:300 |
| RB anti-N-Cadherin | Abcam | Ab18203 | IHC | 1:100 |
| RB anti HA-Probe (Y-11) | Santa Cruz Biotechnology | SC805 | IHC | 1:50 |
| MS anti cardiac (Fetal) Actin | Progen | 61075 | IHC | 1:10 |
| MS anti Desmoplakin | Bio-Rad | 2722-5204 | WB | 1:1000 |
| MS anti Plakoglobin | BD Bioscience | 610235 | WB | 1:1000 |
| RB anti-Desmin | Abcam | Ab15200 | IHC | 1:200 |
| MS anti-y-Cadherin | BD Transduction Laboratories | 610253 | IHC, WB | 1:300 |
| MS anti- Plakophillin2 | Progen | 651101 | IHC, WB | 1:100, 4:10 |
| RB anti Desmocollin2 | Progen | 610120 | IHC | 1:50 |
| MS anti Desmoplakin1&2 | Progen | 651155 | IHC | undiluted |
| RB anti GRP78 BiP | Abcam | Ab21685 | WB | 1:1000 |
| MS anti Desmoglein1&2 | Progen | 61002 | IHC, WB | 1:9, 1:20 |
| RB anti MMP12 | Abcam | Ab66157 | WB | 1 µg / mL |
| Ms anti Galetin3 | Abcam | Ab2785 | WB | 1 µg / mL |
| Rb anti Periostin | Abcam | Ab14041 | IHC/WB | 1:100 / 1 µg / mL |
| Rb anti Smooth Muscle Actin | Abcam | Ab6133 | IHC | 5 µg / mL |
| Rat anti CD11b | Abcam | Ab8878 | IHC | 1:100 |
| Rb anti Vimentin | Abcam | Ab92547 | IHC | 1:100 |
| RB anti -GAPDH | Abcam | Ab181602 | WB | 1:1000 |
| RB IgG-Alexa 488 | Invitrogen | A11034 | IHC | 1:100 |
| RB IgG-Alexa 555 | Invitrogen | A31572 | IHC | 1:100 |
| MS IgG-Alexa 488 | Jackson ImmunoResearch Lab | 715-545-150 | IHC | 1:100 |
| MS IgG- Alexa 555 | Invitrogen | A21422 | IHC | 1:100 |
| MS IgG HRP | GE Healthcare | NXA931 | WB | 1:1000 |
| RB IgG HRP | GE Healthcare | NA934V | WB | 1:1000 |
