# Supplementary material for: Transgenic mice overexpressing desmocollin-2 (DSC2) develop cardiomyopathy associated with myocardial inflammation and fibrotic remodeling
Source: PLoS One. 2017 Mar 24;12(3):e0174019. doi: 10.1371/journal.pone.0174019 (PMC5365111; doi:10.1371/journal.pone.0174019)
Supplement: S3 Table — (DOCX) [file pone.0174019.s005.docx]

**Table S3: Electrocardiogram values.**

|  | P duration [ms] | PR interval [ms] | QRS interval [ms] | QT interval [ms] | QT_c_ [ms] | RR interval | Heart rate [bmp] | |
| --- | --- | --- | --- | --- | --- | --- | --- | --- |
| NT (n=4) | 13.09 ± 2.81 | 34.89 ± 2.77 | 8.58 ± 0.32 | 15.53 ± 1.12 | 50.75 ± 4.57 | 94.91 ± 4.98 | 639.5 ± 31.0 |  |
| TG (n=4) | 11.09 ± 1.37 | 33.22 ± 1.33 | 11.22 ± 0.71 | 30.08 ± 2.45 | 99.50 ± 9.79 | 94.87 ± 9.45 | 644.1 ± 59.5 |  |
| P-value | n. s. | n. s. | <0.05 | <0.05 | <0.05 | n. s. | n. s. |  |

Results are presented as mean ± standard deviation (SD). P-values based on Mann-Whitney test.
